# Buffalo Hospital of the Sisters of Charity

**Published:** 1848-10

**Authors:** 


*Buffalo Hospital of the Sisters of Charity.*—The building, which, for the last two years has been occupied as an Orphan Asylum, has recently been converted into a Hospital, and placed under the management of the Sisters of Charity. Very considerable alterations and repairs have been made, and for a limited number of patients (about one hundred) it has been rendered very comfortable and convenient. The building is located near Main street, a few rods from Virginia Street, being directly in the rear, and closely contiguous to the new edifice in progress of erection for the Medical College. We are happy to state that an arrangement has been made, by which students attending medical lectures at the College, will be admitted, under proper regulations, to the wards of the Hospital, for clinical instruction. The following are the regulations for the reception of patients, &c.:

1. In the admission of patients, no question shall be made as to what the applicant believes on matters of religion; and whenever a patient of any creed may wish to receive spiritual help from the minister of his religion, every facility shall be afforded for having his wishes accomplished.

2. The sick actually suffering from contagious diseases are alone excluded from the Hospital. The medical board will decide the fact of contagion.

3. The Medical board is thus constituted: Frank H. Hamilton, M. D.' attending Surgeon; Austin Flint, M. D. attending Physician; Josiah Trowbridge, M. D., consulting Physician; James P. White, M. D., consulting Surgeon.

4. Applications for admission will be made to the medical board; to the president or vice president of the Good Samaritan Society, or the Society of St. Vincent of Paul; a line from the pastor of any church, of any denomination, will also ensure admission. The persons thus sending a patient will be considered as assuming the charge of paying to the hospital the minimum price for board, attendance, &c.

2. The Sisters are ever willing to admit the sick poor gratis, as far as their means will admit, but as they have no funds, no endowment, they can do but little at present, except to give gratis their time and kindest attentions; hence, the charitable who send a patient, will be responsible to the hospital for patients at the following rates:

1. For the sick poor, in the general ward, for board, washing, &c., medical attendance, nursing, medicines, &c., $1 50 per week.

3. For those in the marine ward, the rate fixed by government.

3. For the sick who can pay for board, medicine, washing, attendance, &c., in a general ward, $2 50 per week.

4. For the sick who are able to pay, and who desire private rooms, the charge will be $4 per week; moderate extras would be required for extraordinary attendance, or costly prescriptions. This class of patients may select any regular physician to attend in conjunction with the attendant physician of the hospital. For medical attendance they will pay their physician according to the rules of the practice.

5. Grossly improper conduct, which cannot be remedied by milder means may cause the expulsion of any patient. The Sisters Servant, after consulting the Board of Directors, will decide on this.

6. Visitors will be admitted from 3 to 5 P. M., every day except Sunday. Relatives of the sick will be admitted at other times, according to the discretion of the Sister Servant, and so as not to harrass the patient.

